# Computational Analysis of Darcy–Forchheimer Flow of Cu/Al–Al_2_O_3_ Hybrid Nanofluid in Water over a Heated Stretchable Plate with Nonlinear Radiation

**DOI:** 10.3390/mi14020338

**Published:** 2023-01-28

**Authors:** Nazek Alessa, R. Sindhu, S. Divya, S. Eswaramoorthi, Karuppusamy Loganathan, Kashi Sai Prasad

**Affiliations:** 1 Department of Mathematical Sciences, College of Sciences, Princess Nourah Bint Abdulrahman University, P.O. Box 84428, Riyadh 11671, Saudi Arabia; 2 Department of Mathematics, Dr. N. G. P. Arts and Science College, Coimbatore 641043, India; 3 Department of Mathematics and Statistics, Manipal University Jaipur, Jaipur 303007, India; 4 Department of Computer Science and Engineering, MLR Institute of Technology, Hyderabad 500043, India

**Keywords:** hybrid nanofluid, magnetic field, non-linear radiation, stretchable plate, Darcy–Forchheimer flow, heat consumption/generation

## Abstract

The aim of this study is to examine the Darcy–Forchheimer flow = of $H_{2}O$-based $Al-Al_{2}O_{3}/$$Cu-Al_{2}O_{3}$ hybrid nanofluid past a heated stretchable plate including heat consumption/ generation and non-linear radiation impacts. The governing flow equations are formulated using the Naiver–Stokes equation. These flow equations are re-framed by using the befitted transformations. The MATLAB bvp4c scheme is utilized to compute the converted flow equations numerically. The graphs, tables, and charts display the vicissitudes in the hybrid nanofluid velocity, hybrid nanofluid temperature, skin friction coefficient, and local Nusselt number via relevant flow factors. It can be seen that the hybrid nanofluid velocity decreased as the magnetic field parameter was increased. The hybrid nanofluid temperature tended to rise as the heat absorption/generation, nanoparticle volume friction, and nonlinear radiation parameters were increased. The surface drag force decreased when the quantity of the magnetic parameter increased. The larger size of the radiation parameter led to enrichment of the heat transmission gradient.

## 1. Introduction

Many scientists and engineers are attempting to improve the heat transmission efficiency since it has an extensive variety of applications in the industrial sectors. Common liquids, such as ethylene glycol, kerosene, water, oil, and polymer-based solutions are used in the heat transmission processes. They have a poor heat transmission rate because of their weaker heat conductivity. To solve this deficiency, experts from several disciplines have attempted to increase the heat conductivity. One of the most effective ways to address this problem is by dispersing nanoparticles across various base fluids. HNFs (hybrid nanofluids) are composed of two or more distinct kinds of nanoparticles in a base fluid. In addition, the HNFs have a heat transmission rate that is much greater than that of general nanofluids, see [1,2,3]. These HNFs may be used in a number of contexts, including in heat exchangers, engine cooling, extrusion processes, micro-manufacturing, drug delivery, energy production, etc. Ikram et al. [4] investigated the flow of $H_{2}O$-based $Ag-TiO_{2}$ hybrid nanofluid in a microchannel. They demonstrated that HNF velocity tended to decrease as HNPVF values increased. The MHD flow of $H_{2}O$-based $Al_{2}O_{3}-Cu$ HNF past a SS was explored by Jawad et al. [5]. They found that the SFC was upgraded when the SVF of the nanoparticles was developing. Devi and Devi [6] elucidated the flow of hydromagnetic $Cu-Al_{2}O_{3}$ HNF in water over a SS. They noticed that the larger HTG occurred in $Cu-Al_{2}O_{3}$ HNF compared to the Cu nanofluid. Shanmugapriya et al. [7] reported on the HMT analysis of HCNT on a wedge with activation energy. They found that the nanoparticle concentration diminished as the NPVF increased. Nayak et al. [8] investigated the slip flow of 3D MHD HNF between parallel plates with entropy optimization. They discovered that the larger Bejan number appeared in HNF compared to the mono nanofluid. The 3D flow of radiative $Cu-Al_{2}O_{3}$ HNF on a shrinking plate was reported by Wahid et al. [9]. They ascertained that the temperature profile improved when the Cu-NPVF improved. Venkateswarlu and Satya Narayana [10] analyzed the MHD flow of $H_{2}O$-based $Cu-Al_{2}O_{3}$ HNF through a porous SS.

Fluid flow via porous medium is a phenomena that occurs in several contexts, including petroleum production, fermentation processes, bio sensors, permeable bearings, electronic boxes, cereal storage, combustion chambers, and casting solidification. A significant amount of work has been done to simulate and study the flow of fluid into porous spaces using Darcy’s law. However, this law is inadequate for larger-velocity and high-porosity conditions. Most physical problems involve greater flow-velocity and stronger-porosity conditions. Forchheimer [11] was able to circumvent this constraint by including a quadratic velocity component in momentum expression. The DFF of HNF on a rotating disk was explained by Haider et al. [12]. They noticed that the larger Forchheimer number causes a reduction in SFC. The Marangoni connective flow of HNF with EG was addresses by Khan et al. [13]. Gul et al. [14] scrutinized the DFF of HNF over a movable thin needle. They noticed that the SFC boomed when the values of the porosity parameter were increased. Alshehri and Shah [15] investigated the radiative DFF of HNF on a parallel SS. They discovered that the larger Forchheimer number caused the increase of HNF temperature. The DFF of HNF across a flat plate was presented by Alzahrani et al. [16]. Sajid et al. [17] discussed the DFF of Maxwell NF past an SS with activation energy. They applied the MATLAB bvp4c solver to solve the governing flow expression numerically. The DFF of non-Newtonian fluid over the Riga plate was inspected by Eswaramoorthi et al. [18]. They found that the fluid speed diminishes when booming the Forchheimer number and porosity parameter.

The heat generation/imbibing processes play a major role in a wide variety of different industrial operations. Some examples are air conditioning, nuclear power plants, boilers, semiconductors, and many others. The impact of the HAG of a HNF over an SS was investigated by Masood et al. [19]. They discovered that the heat generation parameter increases the TBL thickness. The HAG on MHD flow of HNF over an SS was addressed by Zainal et al. [20]. They observed that the HNFT raised when the quantity of HAG parameter increased. The influence of heat production and absorption of an MHD HNF flow past a SS was discussed by Nuwairan et al. [21]. They found that increasing the HAG parameter quantity leads to improvements in the NFT. The rotating flow of $H_{2}O$-based Ag−Cu HNF with HAG was examined by Hayat et al. [22]. They noted that the TBL thickens with a greater size of the HAG parameter. Chalavadi et al. [23] discussed the flow of Carreau/Casson HNF past a moving needle with the HAG effect. They noticed that the HNFT rises with a higher estimation of the HAG parameter. Qayyum et al. [24] discussed the features of HAG of an MHD flow of HNF over an SS. They noticed that the HTG decays when enhancing the HAG parameter. The HT analysis of mono and HNF flow between two parallel plates with HAG was presented by Yaseen et al. [25]. The impact of HAG effects of the flow of CNTs over a SS was analyzed by Zaki et al. [26]. Mishra et al. [27] described the flow of $H_{2}O$-based Ag nanofluid with HAG via a convergent/divergent channel. They found that the HTG strengthens as the heat HAG parameter is improved. The flow of an $H_{2}O$-based $Al_{2}O_{3}-Cu$ HNF with heat absorption and generation was examined by Zainal et al. [28]. Prabakaran et al. [29] developed a mathematical model for the flow of water-based CNTs past an SS with heat consumption/generation. They noted that the greater presence of the HAG parameter decayed the HTG.

The non-linear thermal radiative flow past a stretchable plate is essential in many physical and engineering procedures, including in combustion chambers, atomic plants, aircraft, propulsion devices, power plants, furnace designs etc. Yusuf et al. [30] probed the radiative flow of $Cu-TiO_{2}/H_{2}O$ HNF on a SS with slip condition. They revealed that the EG number quickens when the quantity of the radiation parameter is increased. The MHD NF flow on a plate with radiation was examined by Mustafa et al. [31]. They found that the larger temperature ratio parameter improves the thermal profile. The unsteady 3D MHD flow of HNF with radiation was illustrated by Mabood et al. [32]. They demonstrated that raising the radiation parameter leads to increase the NFT. Kumar et al. [33] explored the radiative flow of Williamson fluid on an SS. They found that the HTG is reinforced when the radiation parameter is improved. The numerical modeling of water-based Ag/Cu NF with radiation was addressed by Qayyum et al. [34]. Patel and Singh [35] investigated the influence of **non-linear radiative** flow of micropolar NF through a non-linear heated SS. Lu et al. [36] scrutinized the MHD flow of Carreau NF over a SS with non-linear radiation. They demonstrated that the TR parameter leads to fortifying the LNN. The influence of non-linear radiative flow of WNF on a SS was probed by Danish Lu et al. [37]. They discovered that by enhancing the radiation parameter causes to decay the local Sherwood number. The MHD flow of Casson HNF past a SS with non-linear radiation was scrutinized by Abbas et al. [38]. Their outcomes show that the temperature distribution escalates with the higher values of the non-linear radiation parameter. Eswaramoorthi et al. [39] investigated 3D radiative flow of CNTs over a Riga plate. They concluded that the Bejan number heightens when improving the radiation parameter.

According to the aforementioned literature reviews, there is still a lack of research on the flow of a $H_{2}O$ based $Al-Al_{2}O_{3}/$$Cu-Al_{2}O_{3}$ HNF past a stretchable plate with convective heating, heat consumption/generation, and non-linear radiation effects. Our research outcomes are used in many numerous technical and industrial applications, like gas turbine rotors, crystal growing, drawing of films, lubrication processes, glider aircraft, power generation, etc.

Finally, the main objective of our investigations is as follows:

- To deliberate the implications of the model’s design on the HNF flow through the stretchable plate.
- How does the usage of HNF lead to affect the velocity and temperature of the fluid?
- How is the HNF temperature impacted by heat generation/absorption and non- linear radiation?
- How is the heat transfer mechanism improved when convective heating conditions are present?

## 2. Mathematical Formulation

**Figure 1:**
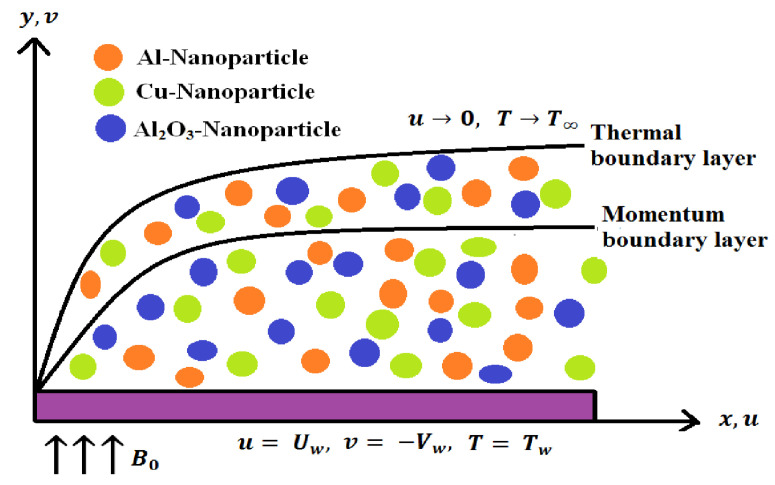
Schematic of the flow model.

The MHD DFF of $H_{2}O$ based $Cu-Al/Al_{2}O_{3}$ HNF past a stretchable plate is investigated. Let *u* and *v* are the HNF velocity factors along the *x* and *y* axes. A stable magnetic field of magnitude $B_{0}$ is activated in the flow direction and resultant magnetic field is disregarded due to small size of Reynolds number. The outcomes of heat generation/absorption and non-linear radiation are also taken into account. Moreover, the sheet and free stream HNFT’s are denoted as $T_{w}$ and $T_{\infty }<T_{w}$, respectively. The physical schematic of the flow model are displayed in Figure 1. The governing mathematical model can be defined as follows based on the preceding assumptions, see Devi and Devi [6]:

(1) $$\begin{array}{ccc} & & \frac{\partial u}{\partial x}+\frac{\partial v}{\partial y}=0 \end{array}$$

(2) $$\begin{array}{ccc} & & u\frac{\partial u}{\partial x}+v\frac{\partial u}{\partial y}=\nu_{hnf}\frac{\partial^{2}u}{\partial y^{2}}-\frac{\nu_{hnf}}{k_{1}}u-\frac{c_{b}}{\sqrt{k_{1}}}u^{2}-\frac{\sigma_{hnf}}{\rho_{hnf}}B_{0}^{2}u \end{array}$$

(3) $$\begin{array}{ccc} & & u\frac{\partial T}{\partial x}+v\frac{\partial T}{\partial y}=\frac{k_{hnf}}{{\left(\rho c_{p}\right)}_{hnf}}\frac{\partial^{2}T}{\partial y^{2}}+\frac{16\sigma^{*}}{3k^{*}{\left(\rho c_{p}\right)}_{hnf}}\frac{\partial }{\partial y}\left(T^{3}\frac{\partial T}{\partial y}\right)+\frac{Q_{0}}{{\left(\rho c_{p}\right)}_{hnf}}\left(T-T_{\infty }\right) \end{array}$$

The initial and boundary conditions are expressed as

(4) $$\begin{array}{ccc} & & u=U_{w}=cx,\;\;v=-V_{w},\;\;T=T_{w}\;\;at\;\;y=0\\ & & u\to 0,\;\;v\to 0,\;\;T\to T_{\infty }\;\;as\;\;y\to 0 \end{array}$$

Define the variables

(5) $$\begin{array}{c} u=cxf^{\prime }\left(\eta \right),\;\;v=-\sqrt{cv_{f}}f\left(\eta \right),\;\;\eta =\sqrt{\frac{a}{\nu_{f}}}y,\;\;\theta =\frac{T-T_{\infty }}{T_{w}-T_{\infty }} \end{array}$$

Implementing the aforementioned adjustments (5) in (2) and (3), we get the following simplified equations:

(6) $$\begin{array}{ccc} & & \frac{1}{A_{1}A_{2}}f^{\prime \prime \prime }\left(\eta \right)+f\left(\eta \right)f^{\prime \prime }\left(\eta \right)-{f^{\prime }}^{2}\left(\eta \right)-Fr{f^{\prime }}^{2}\left(\eta \right)-\lambda f^{\prime }\left(\eta \right)\frac{1}{A_{1}A_{2}}-A_{1}A_{7}Mf^{\prime }\left(\eta \right)=0 \end{array}$$

(7) $$\begin{array}{ccc} & & \frac{A_{5}}{PrA_{3}}\theta \prime \prime \left(\eta \right)+f\left(\eta \right)\theta^{\prime }\left(\eta \right)+\frac{4}{3}\frac{Rd}{PrA_{3}}[{\left(\Gamma -1\right)}^{3}\{\theta^{3}\left(\eta \right)\theta \prime \prime \left(\eta \right)+3\theta^{2}\left(\eta \right){\theta^{\prime }}^{2}\left(\eta \right)\}+{\left(\Gamma -1\right)}^{2}\{3\theta^{2}\left(\eta \right)\theta \prime \prime \left(\eta \right)\\ & & \;\;+6\theta \left(\eta \right){\theta^{\prime }}^{2}\left(\eta \right)\}+\left(\Gamma -1\right)\left\{3\theta \left(\eta \right)\theta^{\prime \prime }\left(\eta \right)+3{\theta^{\prime }}^{2}\left(\eta \right)\right\}+\theta^{\prime \prime }\left(\eta \right)]+Hg\theta \left(\eta \right)\frac{1}{A_{3}}=0 \end{array}$$

The correlated boundary conditions are

(8) $$\begin{array}{c} f\left(0\right)=fw,\;\;f^{\prime }\left(0\right)=1,\;\;\theta \left(0\right)=1,\;\;f^{\prime }\left(\infty \right)=0,\;\;\theta \left(\infty \right)=0 \end{array}$$

where

$$\begin{array}{ccc} & & A_{1}={\left(1-\phi_{1}\right)}^{2.5}{\left(1-\phi_{2}\right)}^{2.5};\\ & & A_{2}=\left(1-\phi_{2}\right)\left(\left(1-\phi_{1}\right)+\phi_{1}\left(\frac{\rho_{1}}{\rho_{f}}\right)\right)+\phi_{2}\left(\frac{\rho_{2}}{\rho_{f}}\right);\\ & & A_{3}=\left(1-\phi_{2}\right)\left(\left(1-\phi_{1}\right)+\phi_{1}\left(\frac{\rho_{1}c_{p1}}{\rho_{f}c_{pf}}\right)\right)+\phi_{2}\left(\frac{\rho_{2}c_{p2}}{\rho_{f}c_{pf}}\right);\\ & & A_{4}=k_{f}\left(\frac{k_{1}+\left(z-1\right)k_{f}-\left(z-1\right)\phi_{1}\left(k_{f}-k_{1}\right)}{k_{1}+\left(z-1\right)k_{f}+\phi_{1}\left(k_{f}-k_{1}\right)}\right);\\ & & A_{5}=\left(\frac{k_{1}+\left(z-1\right)k_{f}-\left(z-1\right)\phi_{1}\left(k_{f}-k_{1}\right)}{k_{1}+\left(z-1\right)k_{f}+\phi_{1}\left(k_{f}-k_{1}\right)}\right)\left(\frac{k_{2}+\left(z-1\right)A_{4}-\left(z-1\right)\phi_{2}\left(A_{4}-k_{2}\right)}{k_{2}+\left(z-1\right)A_{4}+\phi_{2}\left(A_{4}-k_{2}\right)}\right);\\ & & A_{6}=\sigma_{f}\left(\frac{\sigma_{1}+2\sigma_{f}-2\phi_{1}\left(\sigma_{f}-\sigma_{1}\right)}{\sigma_{1}+2\sigma_{f}+\phi_{1}\left(\sigma_{f}-\sigma_{1}\right)}\right);\\ & & A_{7}=\left(\frac{\sigma_{2}+2A_{6}-2\phi_{2}\left(A_{6}-\sigma_{2}\right)}{s_{2}+2A_{6}+\phi_{2}\left(A_{6}-\sigma_{2}\right)}\right)\left(\frac{\sigma_{1}+2\sigma_{f}-2\phi_{1}\left(\sigma_{f}-\sigma_{1}\right)}{\sigma_{1}+2\sigma_{f}+\phi_{1}\left(\sigma_{f}-\sigma_{1}\right)}\right); \end{array}$$

The SFC and the LNN are defined as:

$$\begin{array}{ccc} & & C_{f}\sqrt{Re}=\frac{f\prime \prime \left(0\right)}{A_{1}};\;\;\;\frac{Nu}{\sqrt{Re}}=-\left[A_{5}+\frac{4}{3}Rd{\left(1+\left(\Gamma -1\right)\theta \left(0\right)\right)}^{3}\right]\theta^{\prime }\left(0\right)\; \end{array}$$

## 3. Numerical Solutions

The re-framed expressions (6) and (7) with the correlated boundary restraints (8) are solved numerically by implement the MATLAB bvp4c approach. Initially the higher order problem is transformed into a first order ODE form, see Prabakaran et al. [40]. In this regard, we consider the followings:

$$f=s_{1},f^{\prime }=s_{2},f^{\prime \prime }=s_{3},f^{\prime \prime \prime }={s^{\prime }}_{3},\theta =s_{4},\theta^{\prime }=s_{5},\theta^{\prime \prime }={s^{\prime }}_{5}.$$

$$\begin{array}{ccc} & & {s^{\prime }}_{1}=s_{2}\\ & & {s^{\prime }}_{2}=s_{3}\\ & & {s^{\prime }}_{3}=A_{1}A_{2}\left[{\left(s_{2}\right)}^{2}-s_{1}s_{3}+Fr{\left(s_{2}\right)}^{2}+Ms_{2}A_{1}A_{7}+\lambda s_{2}\frac{1}{A_{1}A_{2}}\right]\\ & & {s^{\prime }}_{4}=s_{5}\\ & & {s^{\prime }}_{5}=\frac{-s_{1}s_{5}-Hgs_{4}\frac{1}{A_{3}}-\frac{4}{3}Rd\frac{1}{A_{3}}\frac{1}{Pr}\left({\left(\Gamma -1\right)}^{3}3{\left(s_{4}\right)}^{2}{\left(s_{5}\right)}^{2}+{\left(\Gamma -1\right)}^{2}6\left(s_{4}\right){\left(s_{5}\right)}^{2}+\left(\Gamma -1\right)3{\left(s_{5}\right)}^{2}\right)}{\left[\frac{A_{5}}{A_{3}}\frac{1}{Pr}+\frac{1}{A_{3}}\frac{1}{Pr}\frac{4}{3}Rd\left[{\left(\Gamma -1\right)}^{3}{\left(s_{4}\right)}^{3}+{\left(\Gamma -1\right)}^{2}3{\left(s_{4}\right)}^{2}+\left(\Gamma -1\right)3\left(s_{4}\right)+1\right]\right]} \end{array}$$

with the constraints are,

$$\begin{array}{ccc} & & s_{1}\left(0\right)=fw,\;\;s_{2}\left(0\right)=1,\;\;s_{4}\left(0\right)=1,\;\;s_{2}\left(\infty \right)=0,\;\;s_{4}\left(\infty \right)=0 \end{array}$$

To solve the above problem numerically, we use the MATLAB bvp4c method with maximal residual error is $10^{-5}$ and size of the step is 0.05.

## 4. Results and Discussion

The primary goal of this section is to delivers the effect of various emerging flow parameters on HNFV, HNFT, SFC and LNN. Table 1 exhibits the thermal properties of aluminum, copper, aluminum oxide, and water. Table 2 shows the mathematical expressions of thermal properties of the HNF. The SFC of water based $Cu-Al_{2}O_{3}$ and $Al-Al_{2}O_{3}$ HNF for various values of *M*, fw, Fr, $\phi_{1}$, $\phi_{2}$ and λ was presented in Table 3. It is perceived that the SFC diminishes when raises the values of Fr, *M*, fw and λ and it improves when strengthening the quantity of $\phi_{1}$ and $\phi_{2}$ for both HNFs. Table 4 presents the LNN for distinct values of Γ, Rd, Hg, fw, Fr and $\phi_{2}$ for both HNFs. It is viewed that the HTR raises when enriching the values of Rd, Γ, fw, and $\phi_{2}$ and the opposite effect attains for the larger size of Hg and Fr for both HNFs. Table 5 exhibits the comparison of θ′(0) with Rd=M=Hg=fw=0 to Devi and Devi [6] for distinct values of Pr and are found in agreeable accord.

**Figure 2:**
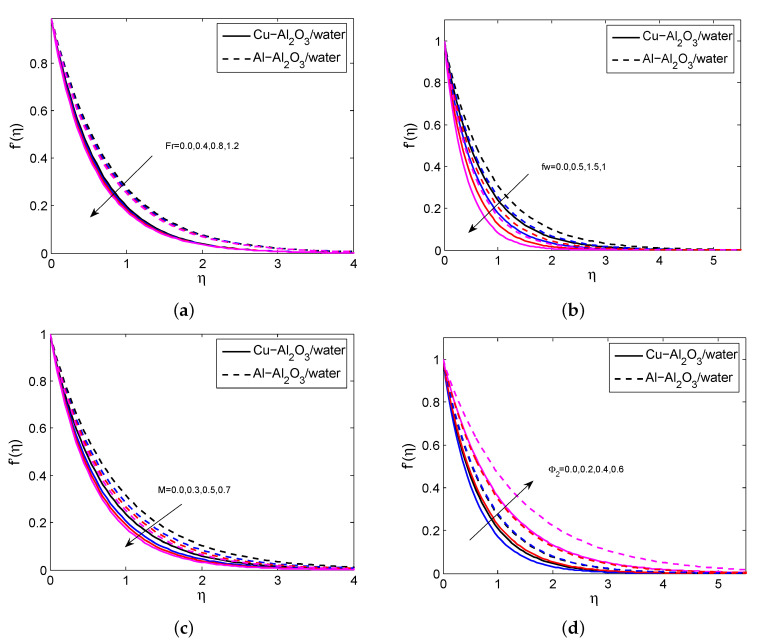
The impact of Fr (**a**), fw (**b**), *M* (**c**), $\phi_{2}$ (**d**) on $f^{\prime }\left(\eta \right)$ for both HNFs.

**Figure 3:**
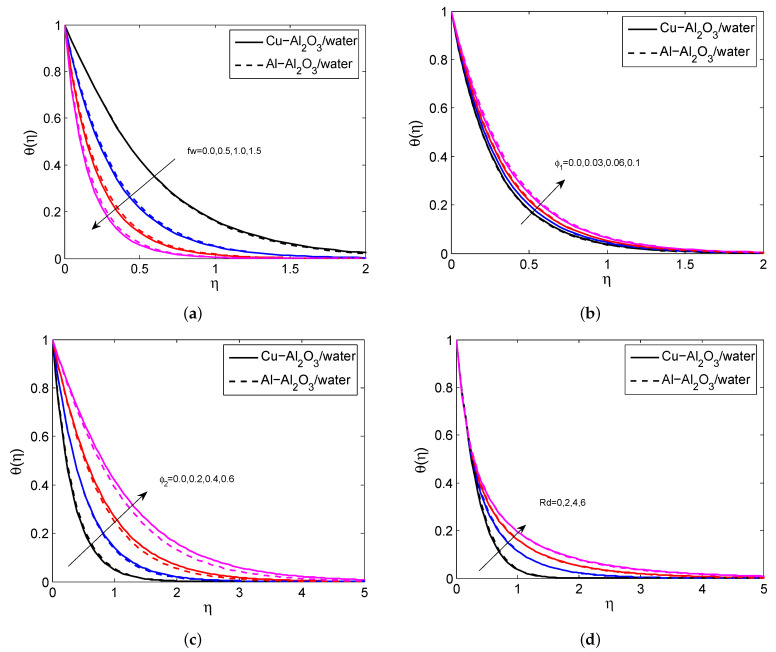
The impact of fw (**a**), $\phi_{1}$ (**b**), $\phi_{2}$ (**c**), Rd (**d**) on θ(η) for both HNFs.

**Figure 4:**
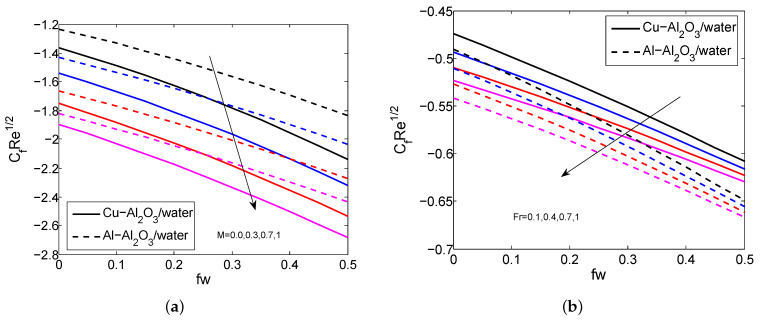
The impact of fw, *M* and Fr on SFC for both HNFs.

**Figure 5:**
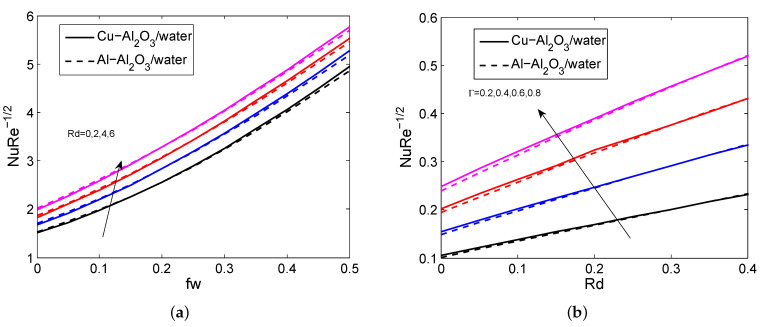
The impact of fw, Rd and Γ on LNN for both HNFs.

Figure 2a–d indicate the influence of Fr, fw, *M*, and $\phi_{2}$ on the HNFV profile. It is believed that the HNFV slumps for the greater values of Fr, fw, and *M* and it aggravates when exalting the values of $\phi_{2}$. Physically, the greater amount of magnetic field creates a drag force called Lorentz force and this force affects the fluid motion. The repercussions of fw, $\phi_{1}$, $\phi_{2}$ and Rd on HNF temperature profile are depicted in Figure 3a–d. It is noticed that the temperature profile grows when enhancing the values of $\phi_{1}$, $\phi_{2}$ and Rd. In contrast, it declines for heightening the values of fw. Physically, as the radiation parameter grows, the HNF’s ability to transfer energy increases, resulting in the growth of the HNFT and the expansion of the TBL. Figure 4a,b shows the impact of *M*, fw and Fr on SFC profile. It is observed that the surface drag force suppresses when the values of *M*, fw and Fr rise. Physically, the improves Lorentz force when it raises the magnetic field, which is affected the movement of fluid flow and thus decreases the surface shear stress. Figure 5a,b depicts the consequences of Rd, fw and Γ on LNN. It is noticed that the HTG improves when enhancing values of Rd, fw and Γ.

**Figure 6:**
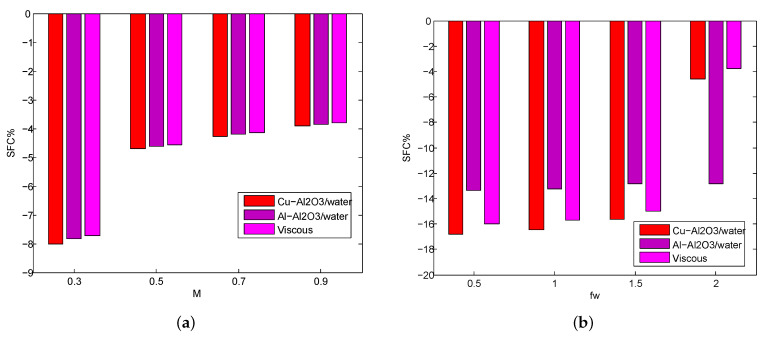

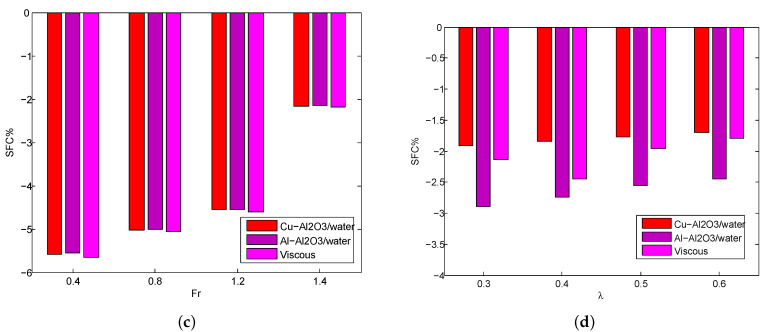
The destructing percentage of SFC for various values of *M* (**a**), fw (**b**), Fr (**c**), λ (**d**) for both HNFs.

Figure 6a–d shows the destructing percentage of SFC for a distinct quantity of M,Fr,fw and λ. In the case of magnetic effect(M), the maximum destructing percentage of SFC is $Cu-Al_{2}O_{3}$ (8.01%), $Al-Al_{2}O_{3}$ (7.84%) and viscous fluid (7.72%) attains when *M* changes from 0 to 0.3 and the minimum destructing percentage of SFC is $Cu-Al_{2}O_{3}$ (3.91%), $Al-Al_{2}O_{3}$ (3.80%) and viscous fluid (3.81%) attains when *M* change from 0.7 to 0.9. In the case of the suction parameter (fw), the maximum destructing percentage of SFC is $Cu-Al_{2}O_{3}$ (16.82%), $Al-Al_{2}O_{3}$ (13.38%) and viscous fluid (16.02%) attains when fw changes from 0 to 0.5 and minimum destructing percentage of SFC is $Cu-Al_{2}O_{3}$ (14.59%), $Al-Al_{2}O_{3}$ (12.23%) and viscous fluid (14.08%) attains when fw changes from 1.5 to 2. In the case of Forchheimer number Fr, the maximum destructing percentage of SFC is $Cu-Al_{2}O_{3}$ (5.58%), $Al-Al_{2}O_{3}$ (5.56%) and viscous fluid (5.64%) attains when Fr changes from 0 to 0.4 and minimum destructing percentage of SFC is $Cu-Al_{2}O_{3}$(2.15%), $Al-Al_{2}O_{3}$ (2.15%) and viscous fluid (2.18%) attains when Fr changes from 1.2 and 1.6. In the case of the porosity parameter (λ), the maximum destructing percentage of SFC is $Cu-Al_{2}O_{3}$ (1.92%), $Al-Al_{2}O_{3}$ (2.91%) and viscous fluid (2.14%) attains when modifies λ from 0.2 to 0.3 and minimal destructing percentage of SFC is $Cu-Al_{2}O_{3}$ (1.70%), $Al-Al_{2}O_{3}$ (2.45%) and viscous fluid (1.88%) attains when modifies Γ from 0.5 to 0.6.

**Figure 7:**
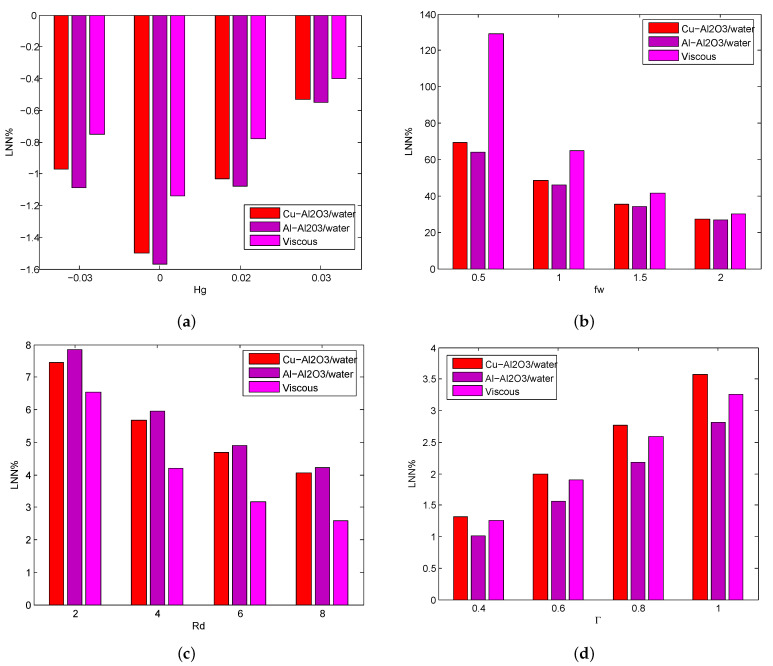
Thedeclining/developing percentage of LNN for various values of Hg (**a**), fw (**b**), Rd (**c**), Γ (**d**) for both HNFs.

The declining/developing percentage of LNN on Hg,fw,Rd, and Γ are portrayed in Figure 7a–d. In the case of heat generation/absorption(Hg), the greatest declining percentage of LNN is $Cu-Al_{2}O_{3}$ (1.49%), $Al-Al_{2}O_{3}$ (1.57%), and viscous fluid (1.14%) attains when Hg changes from −0.03 to 0, and the lowest declining percentage of LNN is $Cu-Al_{2}O_{3}$ (0.53%), $Al-Al_{2}O_{3}$ (0.55%) and viscous fluid (0.40%) attains when Hg changes from 0.02 to 0.03. In the case of suction(fw), the greatest developing percentage of LNN is $Cu-Al_{2}O_{3}$ (69.34%), $Al-Al_{2}O_{3}$ (63.87%), and viscous fluid (129.14%) attains when fw changes from 0 to 0.5 and the lowest developing percentage of LNN is $Cu-Al_{2}O_{3}$ (27.34%), $Al-Al_{2}O_{3}$ (26.69%) and viscous fluid (30.11%) attains when fw changes from 1.5 to 2. In the case of the radiation parameter(Rd), the greatest developing percentage of LNN is $Cu-Al_{2}O_{3}$ (7.45%), $Al-Al_{2}O_{3}$ (7.84%) and viscous fluid (6.53%) attains when Rd changes from 0 to 2 and the lowest developing percentage of LNN is $Cu-Al_{2}O_{3}$ (4.05%), $Al-Al_{2}O_{3}$ (4.23%) and viscous fluid (2.58%) attains when Rd changes from 6 to 8. In the case of the temperature ratio parameter (Γ), the greatest developing percentage of LNN is $Cu-Al_{2}O_{3}$ (3.57%), $Al-Al_{2}O_{3}$ (2.81%) and viscous fluid (3.26%) attains when Γ changes from 0.8 to 1 and the lowest developing percentage of LNN is $Cu-Al_{2}O_{3}$ (1.31%), $Al-Al_{2}O_{3}$ (1.02%) and viscous fluid (1.26%) attains when Γ changes from 0.0 to 0.4.

## 5. Conclusions

The steady, 2D, non-linear radiative Darcy-Forchheirmer flow of $H_{2}O$ based hybrid nanofluid past a stretchable plate with the presence of heat absorption/generation and magnetic field was investigated. Two different mixture of hybrid nanofluid, namely $Cu-Al_{2}O_{3}$ and $Al-Al_{2}O_{3}$ are taken into account. The governing flow models are re-changed by implementing the suitable transformations and solved by using MATLAB bvp4c code. Some remarkable observations of our findings are given below.

- The hybrid nanofluid velocity profile decrepitude’s for larger quantity of Fr (Forchheirmer), *M* (magnetic field parameter) and fw (suction/injection parameter).
- The larger values of Rd (radiation parameter) improve the hybrid nanofluid fluid temperature.
- The hybrid nanofluid has a larger heat transfer rate than the ordinary fluid.
- The more presence Fr (Forchheirmer number), *M* (magnetic field parameter) and fw (suction/injection parameter) causes to reduce the skin friction coefficient.
- The Rd (radiation parameter) and Γ (temperature ratio parameter) lead to enriching the heat transfer rate.
- The $Cu-Al_{2}O_{3}$ hybrid nanofluid have higher heat transfer rate than the $Al-Al_{2}O_{3}$ hybrid nanofluid.

## Figures and Tables

**Table 1:** The thermal properties of $H_{2}O$, Cu, Al and $Al_{2}O_{3}$.

Physical Properties	Fluid Phase $\left(H_{2}O\right)$	Copper (Cu)	Aluminum (Al)	Aluminum Oxide $\left({\mathrm{Al}}_{2}O_{3}\right)$
ρ (kg/m${}^{3}$)	997.1	8933	2719	3970
$c_{p}$ (J/kgK)	4179	385	903	765
*k* (W/mk)	0.613	400	237	40

**Table 2:** Thermophysical properties of Hybrid nanofluid.

Properties	Hybrid Nanofuid
Density	$\rho_{hnf}=\left(1\;-\;\phi_{2}\right)\left[\left(1\;-\;\phi_{1}\right)\rho_{f}\;+\;\phi_{1}\rho_{s1}\right]\;+\;\phi_{2}{\left(\rho c_{p}\right)}_{s2}$
Heat Capacity	${\left(\rho c_{p}\right)}_{hnf}=\left(1\;-\;\phi_{2}\right)\left[\left(1\;-\;\phi_{1}\right){\left(\rho c_{p}\right)}_{f}\;+\;\phi_{1}{\left(\rho c_{p}\right)}_{s1}\right]\;+\;\phi_{2}{\left(\rho c_{p}\right)}_{s2}$
Viscosity	$\mu_{hnf}=\frac{\mu_{f}}{{\left(1\;-\;\phi_{1}\right)}^{2.5}({\left(1\;-\;\phi_{2}\right)}^{2.5}}$
Thermal conductivity	$\frac{k_{hnf}}{k_{bf}}=\frac{k_{s2}\;+\;\left(n\;-\;1\right)k_{bf}\;-\;\left(n\;-\;1\right)\phi_{2}\left(k_{bf}\;-\;k_{s2}\right)}{k_{s2}\;+\;\left(n\;-\;1\right)k_{bf}\;+\;\phi_{2}\left(k_{bf}\;-\;k_{s2}\right)}$
	$where\frac{k_{bf}}{k_{f}}=\frac{k_{s1}\;+\;\left(n\;-\;1\right)k_{f}\;-\;\left(n\;-\;1\right)\phi_{1}\left(k_{f}\;-\;k_{s1}\right)}{k_{s1}\;+\;\left(n\;-\;1\right)k_{f}\;+\;\phi_{1}\left(k_{f}\;-\;k_{s1}\right)}$
Electrical conductivity	$\frac{\sigma_{hnf}}{\sigma_{bf}}=\frac{\sigma_{s2}\;+\;2\sigma_{bf}\;-\;2\phi_{2}\left(\sigma_{bf}\;-\;\sigma_{s2}\right)}{\sigma_{s2}\;+\;2\sigma_{bf}\;+\;\phi_{2}\left(\sigma_{bf}\;-\;\sigma_{s2}\right)}$
	$\frac{\sigma_{bf}}{\sigma_{f}}=\frac{\sigma_{s1}\;+\;2\sigma_{bf}\;-\;2\phi_{1}\left(\sigma_{bf}\;-\;\sigma_{s1}\right)}{\sigma_{s1}\;+\;2\sigma_{bf}\;+\;\phi_{1}\left(\sigma_{1}\;-\;\sigma_{s1}\right)}$

**Table 3:** The SFC for different values of *M*, fw, Fr, $\phi_{2}$, λ and $\phi_{1}$ for both HNFs.

						SFC	
M	fw	Fr	$\phi_{2}$	λ	$\phi_{1}$	$\mathrm{Cu}-{\mathrm{Al}}_{2}O_{3}$	$\mathrm{Al}-{\mathrm{Al}}_{2}O_{3}$
0	0.4	0.4	0.04	0.2	0.1	−1.041285	−0.828107
0.3						−1.131997	−0.898513
0.5						−1.187911	−0.942100
0.7						−1.240878	−0.983495
0.9						−1.291330	−1.023007
0.5	0	0.4	0.04	0.2	0.1	−1.025049	−0.839781
	0.5					−1.232296	−0.969511
	1					−1.475034	−1.117201
	1.5					−1.748714	−1.281414
	2					−2.047345	−1.460010
0.5	0.4	0	0.04	0.2	0.1	−1.121572	−0.889696
		0.4				−1.187911	−0.942100
		0.8				−1.250736	−0.991766
		1.2				−1.310531	−1.039069
		1.4				−1.339416	−1.061929
0.5	0.4	0.4	0.005	0.2	0.1	−1.234564	−1.017737
			0.02			−1.217739	−0.986006
			0.04			−1.187911	−0.942100
			0.06			−1.151153	−0.896874
			0.08			−1.108898	−0.850821
0.5	0.4	0.4	0.04	0.2	0.1	−1.187911	−0.942100
				0.3		−1.211098	−0.970320
				0.4		−1.233775	−0.997638
				0.5		−1.255976	−1.024136
				0.6		−1.277729	−1.049886
0.5	0.4	0.4	0.04	0.2	0.1	−1.187911	−0.942100
					0.2	−0.823882	−0.580253
					0.3	−0.530334	−0.340609
					0.4	−0.313293	−0.189744
					0.5	−0.166711	−0.099836

**Table 4:** The LNN for different values of Γ, Rd, Hg, fw, Fr, $\phi_{2}$ for both HNFs.

						LNN	
Γ	Rd	Hg	fw	Fr	$\phi_{2}$	$\mathrm{Cu}-{\mathrm{Al}}_{2}O_{3}$	$\mathrm{Al}-{\mathrm{Al}}_{2}O_{3}$
0.2	0.6	−0.05	0.4	0.4	0.04	4.612967	3.718700
0.4						4.673327	3.756509
0.6						4.766403	3.815178
0.8						4.898593	3.898462
1						5.073613	4.008004
0.1	0	−0.05	0.4	0.4	0.04	4.482528	4.336498
	2					4.816646	4.676659
	4					5.090079	4.955152
	6					5.329128	5.198584
	8					5.545070	5.418359
0.1	0.6	−0.05	0.4	0.4	0.04	3.857127	3.705975
		−0.03				3.819692	3.668231
		0				3.762517	3.610602
		0.03				3.704058	3.551706
		0.04				3.704058	3.531735
0.1	0.6	−0.05	0	0.4	0.04	2.978466	2.966152
			0.5			5.043729	4.860771
			1			7.493146	7.097396
			1.5			10.15220	9.527814
			2			12.92796	12.07080
0.1	0.6	−0.05	0.4	0	0.04	4.602380	4.456895
				0.4		4.592433	4.448303
				0.8		4.583213	4.440315
				1.2		4.574618	4.432844
				1.4		4.570526	4.429281
0.1	0.6	−0.05	0.4	0.4	0.005	4.500213	4.353271
					0.02	4.539167	4.393577
					0.04	4.592433	4.448303
					0.06	4.647299	4.504242
					0.08	4.703862	4.561491

**Table 5:** Comparison of $-\theta^{\prime }\left(0\right)$ at different values of Pr with $Rd=\phi_{1}=\phi_{2}=Hg=\Gamma =Fr=fw=0$, see Devi and Devi [6].

	−θ′(0)	
***Pr***	**Devi and Devi [6]**	**Present Results**
2.00	0.91135	0.911358
6.13	1.75968	1.759687
7.00	1.89540	1.895407
20.0	3.35390	3.353952

## Data Availability

Not applicable.

## Nomenclature

Symbols	Description
x,y	Cartesian coordinates (m)
θ	dimensionless temperature
$c_{b}$	drag coefficient
$Q_{0}$	heat consumption/generation coefficient (W m${}^{-3}$ K${}^{-1}$)
$\frac{Nu}{\sqrt{Re}}$	local Nusselt number
$k_{1}$	permeability of porous medium
$k^{*}$	Rosseland absorption coefficient
$C_{f}\sqrt{Re}$	skin friction coefficient
$T_{w}$	surface temperature (K)
$\tau_{w}$	surface shear stress
$U_{w},V_{w}$	surface stretching velocities (m${}^{2}$ s${}^{-1}$)
fw	suction/injection parameter
$c_{p}$	specific heat capacity
T	temperature of the fluid (K)
$T_{\infty }$	temperature away from the plate (K)
u,v	velocity components
$Fr\left(=\frac{xc_{b}}{\sqrt{k_{1}^{*}}}\right)$	Forchheimer number
$\Gamma \left(=\frac{T_{w}}{T_{\infty }}\right)$	temperature ratio parameter
$Hg\left(=\frac{Q}{{\left(\rho c_{p}\right)}_{nf}c}\right)$	heat consumption/generation parameter
$M\left(=\frac{\sigma B_{0}^{2}}{\rho_{f}a}\right)$	magnetic field parameter
$\lambda \left(=\frac{\nu_{f}}{k^{*}c}\right)$	porosity parameter
$Pr\left(=\frac{{\left(\mu c_{p}\right)}_{f}}{k_{f}}\right)$	Prandtl number
$Rd\left(=\frac{4\sigma T_{\infty }^{3}}{k^{*}k_{f}}\right)$	Radiation parameter
Greeksymbols	
ρ	density
$\rho_{nf}$	density of nanofluid
$\rho_{hnf}$	density of hybrid nanofluid
σ	electrical conductivity
$\sigma_{nf}$	electrical conductivity of nanofluid
$\sigma_{hnf}$	electrical conductivity of hybrid nanofluid
ν	kinematic viscosity
$\sigma^{*}$	Stefan-Boltzmann constant
μ	viscosity
$\mu_{nf}$	viscosity of nanofluid
$\mu_{hnf}$	viscosity of hybrid nanofluid

## Abbreviations

CNTs	carbon nanotubes
DFF	Darcy-Forchheimer flow
EG	entropy generation
HAG	heat absorption/generation
HMT	heat mass transfer
HT	heat transfer
HTG	heat transfer gradient
HNF	hybrid nanofluid
HCNT	hybrid carbon nanotube
HNPVF	hybrid nanoparticle volume fraction
LNN	local Nusslet Number
MHD	magneto-hydro-dynamics
NF	nanofluid
NFT	nanofluid temperature
NFV	nanofluid velocity
NLR	non-linear radiation
NPVF	nanoparticle volume fraction
SS	stretching sheet/surface
SFC	skin friction coefficient
SVF	solid volume fraction
TBL	thermal boundary layer
TR	temperature ratio
WNF	Williamson nanofluid
